# Root Canal Infection and Its Impact on the Oral Cavity Microenvironment in the Context of Immune System Disorders in Selected Diseases: A Narrative Review

**DOI:** 10.3390/jcm12124102

**Published:** 2023-06-17

**Authors:** Jarosław Sobieszczański, Sebastian Mertowski, Katarzyna Sarna-Boś, Piotr Stachurski, Ewelina Grywalska, Renata Chałas

**Affiliations:** 1 Preclinical Dentistry Lab, Medical University of Lublin, Chodźki 6 Street, 20-093 Lublin, Poland; jaroslawsobieszczanski@umlub.pl; 2 Department of Experimental Immunology, Medical University of Lublin, 4a Chodźki Street, 20-093 Lublin, Poland; ewelina.grywalska@umlub.pl; 3 Department of Dental Prosthetics, Medical University of Lublin, Chodźki 6 Street, 20-093 Lublin, Poland; katarzynasarnabos@umlub.pl; 4 Department of Pediatric Dentistry, Medical University of Lublin, 20-093 Lublin, Poland; piotr.stachurski@umlub.pl; 5 Department of Oral Medicine, Medical University of Lublin, Chodźki 6 Street, 20-093 Lublin, Poland; renatachalas@umlub.pl

**Keywords:** root canal, additional root canals, inflammation, immune system

## Abstract

The oral cavity has a specific microenvironment, and structures such as teeth are constantly exposed to chemical and biological factors. Although the structure of the teeth is permanent, due to exposure of the pulp and root canal system, trauma can have severe consequences and cause the development of local inflammation caused by external and opportunistic pathogens. Long-term inflammation can affect not only the local pulp and periodontal tissues but also the functioning of the immune system, which can trigger a systemic reaction. This literature review presents the current knowledge on root canal infections and their impact on the oral microenvironment in the context of immune system disorders in selected diseases. The result of the analysis of the literature is the statement that periodontal-disease-caused inflammation in the oral cavity may affect the development and progression of autoimmune diseases such as rheumatoid arthritis, systemic lupus erythematosus, or Sjogren’s syndrome, as well as affecting the faster progression of conditions in which inflammation occurs such as, among others, chronic kidney disease or inflammatory bowel disease.

## 1. Introduction

The oral cavity, due to its function related to the intake and processing of food, is a place with a very diverse microenvironment, affecting the structures within it, e.g., teeth [1]. These structures are constantly exposed to mechanical damage as well as chemical damage, to which the oral microbiome contributes [2,3,4,5]. In the case of the structure of teeth, their sensitive part, which is the pulp and the root canal system, is an intact place to which both pathogens and commensal bacteria in the oral cavity should not have access [6,7]. When the dentinal barriers are damaged by caries, fissures, or trauma, both opportunistic and pathogenic microorganisms gain access to the pulp chamber and root canal system to which they should never have access [8,9,10]. This situation could lead to harmful effects such as inflammation, pulp necrosis, and periodontitis [11,12,13]. Even without symptoms, long-term inflammation within the tooth’s apex and necrotic pulp can develop over many years [14]. These chronic inflammatory changes can not only affect local tissue but also the immune system, leading to the development of related diseases. This literature review aimed to present the current state of knowledge in the field of tooth root canal infections and its impact on the oral cavity microenvironment in the context of disorders of the immune system in selected diseases.

## 2. Differentiation of Root Canals and Their Participation in Signaling Pathways

### 2.1. Differentiation of Root Canals

As scientific research shows, different types of teeth (incisors, canines, premolars, and molars) will have different numbers of canals: incisors and canines will have one canal, premolars will have one or two, and molars will have three or more. A root canal system contains the pulp or living tissue inside the tooth. What is more, anatomical differences between patients, as well as the possibility of additional canals that extend from the main root canals, effectively prevent a comprehensive approach to therapy. Accessory canal ducts can cause problems because they can be small, narrow, and difficult to detect via X-ray. Furthermore, even if the dentist finds an additional root canal, it may be difficult to access and clean it, which means that infection may remain, requiring another root canal procedure. That is why, as researchers suggest, it is so important to develop new procedures for visualizing and cleaning the root canal system, and reaching the smallest, additional canals is often not possible. This can leave small amounts of organic material in these hard-to-reach places, which can lead to the development of infection [15].

We can distinguish a wide range of different variations in root canal anatomy, ranging from different root canal configurations, developmental anomalies, and morphologies of smaller canals, including accessory canals and apical deltas. However, the terminology for accessory canals seems to be inconsistent [16]. From early reports, such as DeDeus (1975) [17], we can find a division of such canals into three terms: side canal, secondary canal, and finally, additional canal. A lateral canal is a canal that starts in the main root canal and reaches the periodontium in the root body. The secondary canal is defined similarly to the previous one, only its location is in the apical region. According to this terminology, the accessory canal is the one that emerges from the secondary canal, reaching the periodontal ligament, and that is located in the apical part of the root [17]. Other authors use the terms reticular, recurrent, or auxiliary to describe canals other than the main canals [18]. Some authors, such as Cheung, have defined the difference between the lateral canal and the secondary canal based on the angle of these canals from the main canals. Thus, the branches extending at right angles were called lateral canals, and those extending obliquely into the periodontal space were called additional canals [19]. For other authors, lateral canals were types of canals located in different parts of the roots (coronal, middle, and apical) [20]. Various terms describing accessory canals and their orifices can be found in the American Association of Endodontists’ Glossary of Endodontic Terms, updated in March 2020 [21].

An accessory canal is any branch of the main canal or pulp chamber that communicates with the outer surface of the root. The lateral canal, on the other hand, is a kind of additional canal, an additional canal located in the coronal or middle third of the root, usually extending horizontally from the space of the main canal. Additionally, according to AAE, the furcation canal is an accessory canal located in the furcation [21]. A very special branching point of the canal is the apical part of the root. At the end of the root canal, the apical delta can be localized. The definition of an apical delta, according to the AAE Glossary of Endodontic Terms, is the morphology of the pulp canal in which the main canal divides into many accessory canals near the apex [21]. Historically, in the early work of Hess in 1917, it was noted that the root canal system includes branches of the main root canal [22]. It was Weine et al. who first provided a clinical classification of root canal configurations with their study of the mesiobuccal root canals of the maxillary first molars [22,23].

**Figure 1:**
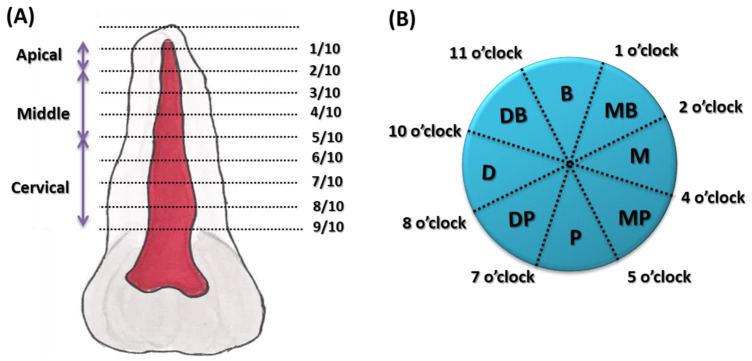
Classification by Yoshiuchi et al. (**A**) Dividing the root length into ten parts; (**B**) orientation of the accessory canals with numeration on the cross-section as on a clock face [27].

This initial and still very important classification was developed by Vertucci, and four more possible configurations were added. Finally, eight possible configurations were presented [24]. In the following years of research on this topic, Vertucci’s classification still needed to be more comprehensive, and subsequent scientists developed it even more. Gulabivala et al., examining mandibular molars, added seven more possible configurations to Vertucci’s classification [25]. Sert and Bayirli used the debridement technique to assess root canal configurations in 2800 teeth and added a further fourteen new root canal configurations to the Vertucci classification [26]. All the scientific studies cited here show the development of the root canal configuration classification systems and research techniques. This pushes the systematic development, and encourages more and more thorough study, of the root canal system in human teeth. In turn, the classification of accessory canals in terms of their morphology was initiated by the work of Yoshiuchi et al. The classification was based on the location of the secondary canal along the root and divided the length of the root into 10 parts, starting from the apex (1st part) through the middle (2nd to 4th part) to the cervical (5th to 9th part) location (Figure 1A). The orientation of the accessory canals was also numbered by the authors on the cross-section as on a clock face, where noon corresponds to the middle of the buccal or labial surface, and six o’clock corresponds to the central lingual or palatal surface of the root [27] (Figure 1B).

Another classification was proposed by Vertucci (1974), who divided the lateral canals into four groups depending on their location: coronal, middle, apical, and furcation [24]. The furcation region, as already mentioned, is a very special place in the root canal system. In 1975, Yoshida et al. focused on this area of the tooth and proposed the classification of additional canals in the area of the furcation [15]. The main idea behind this classification was to observe the six possible configurations and compile them into six types (Table 1).

To supplement Weine’s classification of root canal morphology, Matsunaga et al. (2014) showed a different classification related to Weine’s [28]. The authors divided each type of root canal configuration into four distinct subtypes, but without any information about number, localization, or configuration. This classification into four groups was as follows:

I. no accessory canals;

II. with apical ramifications;

III. with lateral canals;

IV. apical ramifications and lateral canals were observed simultaneously [28,29].

Finally, Ahmed et al. (2018) proposed a new classification system for accessory canal morphology [16]. This was the answer to previous attempts, which did not fulfill the complete need for simple, practical, and precise classification, including the number, location, and configuration of accessory canals.

**Figure 2:**
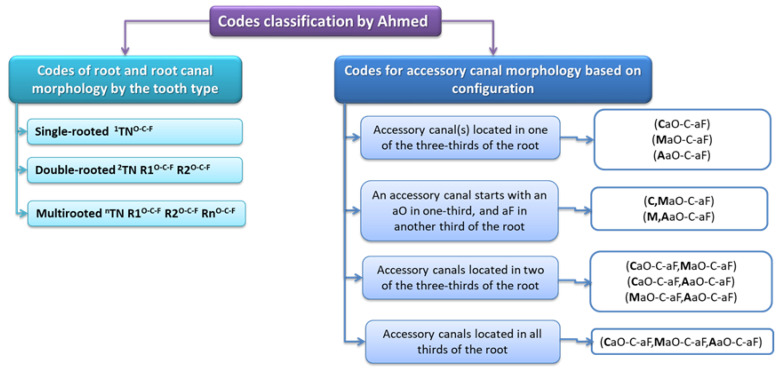
Classification of accessory canals by Ahmed et al. [16].

In this classification, root canal length is divided into three parts: coronal (C), middle (M), and apical (A). The complete description of the accessory canals in a tooth consists of a code in which the first digit in the superscript is the number of separate roots, then the tooth number, followed by the root number, followed by the superscript, with the number of accessory canals in parentheses being given with their location. It is quite a complicated code, but it gives precise information about the number and localization of accessory canals [16] (Figure 2).

### 2.2. Communication Pathways between the Pulp and Periodontal Tissues

To better understand the role of inflammation and its impact on the immune system, it is important to understand the role of communication pathways between the pulp and periodontal tissues. The pulp and periodontal tissues are closely interconnected through the various possible communication media. Communication pathways may be located in different parts of the root canal and pulp chamber. These pathways facilitate the transmission of biological, chemical, and thermal injuries. The apical foramen and accessory canals are the best-known and basic anatomical connections (e.g., lateral and furcation) [30]. These communication pathways are essential for vascular, neural, and connective tissue connectivity [16]. However, these soft tissue connections are not the only means of communication between the pulp and periodontium. The exposed cervical dentine, not covered by either cement or enamel, also provides tubular communication through the dentinal tubules. This type of connection also occurs in physiological root resorption [16,30,31,32,33,34]. Even cementum itself does not provide perfect tightness. The permeability of cement has been described and proven in various early studies, e.g., by Wasserman et al. [35].

It should also be noted that anatomical communication undergoes dynamic changes during the life of the human body. It changes with the age of the body and is subject to various physiological mechanisms. With age, the apical foramen decreases in diameter and deviates from the long axis of the root due to cement deposition. Gradually, the number of additional canals decreases, and the permeability of the root dentine decreases. These physiological mechanisms lead to a gradual occlusion of the dentinal tubules, especially in the apical part of the root [36,37]. Cement as a tissue also becomes less permeable with age [37]. In addition to physiological pulp–periodontal connections, one should not forget about the possible and proven pathological connections. Such pathological paths include fractures, cracks, and perforations of the tooth, in particular of the root. These are direct connections. In addition to these, there are also indirect connections, such as deep root fissures, which can cause periodontal thinning, thus allowing the irritant to pass through the tooth structure. The variety and variability of physiological and pathological communication pathways between the pulp and periodontal tissues indicate a very complex relationship between these two types of tissues [31,33,34,35,36].

## 3. Interactions between the Immune System and Root Canals

To fully understand the interactions that occur between the immune system and root canals, we need to understand how the oral microbiota are shaped and how microorganisms can colonize root canals. Scientific research estimates that over 700 different species of bacteria can be distinguished in the oral cavity, including *Streptococcus* mutants, *Enterococcus* spp., *Peptostreptococcus* spp., *Actinomyces* spp., and *Porphyromonas gingivalis*, as well as numerous fungi, e.g., *Candida albicans* and protozoa (Entamoeba gingivitis) or viruses (Cytomegalovirus, herpes virus) [38,39,40]. Such a diverse oral microbiome constantly affects all structural elements in the oral cavity, including teeth. Despite the unique structural features of teeth (hard tissues, enamel, dentin), microorganisms have found a way to penetrate inside, leading to root canal infection. One of the mechanisms of violating the hard tissues of the tooth is the secretion of compounds by microorganisms that allow the hydroxyapatite to dissolve or penetrate it through cracks in the tooth crown or exposed tubules. They also often penetrate as a result of the development of caries or damage caused by dental procedures (cavity preparation or cytotoxic effect of dental materials) [41,42]. The pulp is a tissue capable of reacting to all kinds of stimuli, including injuries, thermal or chemical irritations, and the penetration of pathogenic bacteria that activate the immune response. The development of inflammation, which is a consequence of the penetration of pathogenic microorganisms into the pulp, is caused by their by-products (e.g., bacterial toxins) or structural elements (lipoteichoic acid, lipopolysaccharide) that penetrate the dentinal tubules [43,44]. The prolonged presence of microorganisms leads not only to inflammation of the pulp but also infection of the root system or periapical tissues, which may cause periapical diseases [45,46,47].

Endodontito inflammation of the pulp is due to the ability of pathogenic microorganisms to form biofilms, i.e., these structures provide bacteria with protection against other microorganisms, antimicrobial agents, and host defense mechanisms. Biofilm formation provides microbes with a more favorable ecological niche and promotes increased metabolic activity and, thus, increased pathogenicity [11,48,49]. Studies have shown that the biofilm created by microorganisms can be inside the roots (up to 77% of all cases) or outside the root (6% of all cases) [50,51,52]. Therefore, how does the immune system work in such conditions?

As a result of the development of inflammation of the pulp, pro-inflammatory mediators (especially cytokines) are overexpressed in pulpal fibroblasts, and there is an increased expression of receptors recognizing molecular patterns of pathogens (PAMPs) on fibroblasts and odontoblasts, which are part of the innate immune response, which is the first line of defense against pathogens [53,54,55]. Pattern recognition receptors (PRRs) can also be expressed by immune cells such as macrophages, monocytes, neutrophils, or dendritic cells, or antigen-specific cells such as T and B lymphocytes [56,57,58]. Cells capable of phagocytosis are an extremely important element of the innate immune response because, as a result of direct interaction, they can remove encountered pathogens by phagocytosis, as well as being responsible for the production of inflammatory mediators (cytokines and chemokines), which are involved in the recruitment of other immune cells to the site of infection [59,60,61,62,63,64,65,66,67,68,69].

The expression of PRRs, which include Toll-like receptors (TLRs), on host cells allows the recognition of specific pathogens. It thus confers a degree of specificity to the innate immune system [63,64,65,66,67,68,69,70]. Numerous scientific studies confirm this. In the works by Durand et al. and Pääkkönen et al., odontoblasts have been shown to express TLR1–TLR6 and TLR9 but not TLR7, TLR8, and TLR10 [64,65]. The activation of TLR2 by lipoteichoic acid (LTA) induces the production of pro-inflammatory cytokines. It is also associated with the increased ability of odontoblasts not only to recognize but also to respond to many antigens, which are most often by-products of bacterial and viral metabolism [66,67]. Recent studies have shown that pulpal fibroblasts can upregulate the expression of some TLRs. This was confirmed by the teams of Jiang et al. in 2006, and Keller et al., and Park et al. in 2010, whose studies showed the increased expression of TLR2-TLR5 in human pulpal fibroblasts [66,67,68]. The second type of PRRs is the NLRs (NOD-like receptors), which are also involved in the host response to bacterial pulp infection. The intracellular NOD1 and NOD2 receptors participate in the innate immune response through the NF-κB pathway, which induces the expression of pro-inflammatory mediators such as IL-8. Studies have shown that both types of receptors are expressed in normal tooth pulp and are upregulated in inflammatory reactions [67,69].

Research conducted by Keller et al. in 2010, and Lee et al. in 2014, showed that NOD2 was involved in odontoblast differentiation (through the MAPK pathway) and osteoclastogenesis (through M-CSF and RANKL) [69,71]. Research conducted by Zhang et al. in 2015 drew the attention of researchers to yet another pathogen recognition mechanism involving the NALP3 inflammasome. It is the best-characterized inflammasome in recent years, which can be activated by many stimuli, including PAMP and DAMP, but also by all pathogens or environmental factors. NALP3 is expressed in human dental pulp cells, inflammatory cells, and pulp fibroblasts. In their research, Zhang’s team showed that the activation of NALP3 due to the response to bacterial LPS causes the activation of the TLR4/NF-κB pathway and the secretion of IL-1β [72]. PRR expression is not limited only to the pulp cells; their excessive activation is also observed in macrophages, dendritic cells, and T and B lymphocytes. The inflammatory changes initiated by multifactorial infections and their metabolism products can lead to tissue destruction, bone destruction, root resorption, the development of diseases within the oral cavity, and systemic and autoimmune diseases [73,74].

This section may be divided by subheadings. It should provide a concise and precise description of the experimental results, their interpretation, as well as the experimental conclusions that can be drawn.

### 3.1. Chronic Apical Periodontitis

Periodontitis is a disease in which the tissues surrounding the tooth, i.e., the periodontium, bone, and gums, are destroyed. The main factor of periodontitis is bacteria living in plaque and periodontal pockets. Risk factors for the disease include stress, certain medications, smoking, and genetic factors [75,76].

Chronic apical periodontitis is an extremely dynamic type of root canal infection. This infection is caused by persistent, localized inflammation within the periapical tissue, which can lead to progressive bone resorption and the formation of periapical lesions. The main reason for this is the presence of bacteria that remain in the root canal system, specifically in the dentine tubules, the apical delta of the lateral canals, and their branches [77,78,79]. As a result of pulp destruction, a lack of blood supply, and the presence of pathogenic microorganisms, inflammation develops and is maintained. As a result of chronic inflammation, both the body’s innate and adaptive immune responses are activated, resulting in the recruitment of various types of cells and inflammatory mediators, which then lead to the destruction of the periapical tissue and the formation of periapical lesions [80].

The chronic foci of infection are responsible for the increased resistance of microorganisms to antibacterial agents, which makes it difficult to use an effective therapeutic process. Therefore, eliminating microorganisms from the infected root canal system, including hard-to-reach additional canals, requires introducing a modern strategy of endodontic antibiotic therapy, including thorough antiseptic treatment with drugs and implementing modern technologies for the instrumental treatment of root canals [81].

### 3.2. Acute Apical Abscess

Another oral disease associated with root canal infection is the occurrence of an acute dental abscess. Its formation is closely related to the occurrence of trauma, the development of caries, and unsuccessful endodontic treatment. Root canal colonization occurs due to the pulp chamber being breached, where mainly anaerobic microorganisms, capable of creating a biofilm, enter and develop [82,83].

The development of the abscess itself is due to the entry of bacteria or their toxins through the apical foramen into the periapical tissues, which causes acute inflammation and pus formation. Studies show that most dental abscesses respond to antibiotic therapy but, in some patients, surgical treatment of the infection may be necessary [82,84]. The research results presented by the Böttger team showed that odontogenic infections are multifactorial and mainly caused by anaerobic bacteria strains, with aerobic bacteria and facultative anaerobes playing a minor role in the pathogenesis. The microbiome of oil from patients they analyzed showed an extremely diverse quantitative and qualitative composition of colonizing microorganisms. Some cases showed a monoinfection caused by *Streptococcus* spp. (accounting for 99% of all detected microorganisms) or a typical picture of a multibacterial infection (*Prevotella* spp., *Streptococcus* spp., *Lactobacillus* spp., *Rothia* spp., *Veillonella* spp.) [85].

## 4. The Spread of Endodontic Bacteria to Adjacent Tissues and Organs, or How Systemic Infections and the Development of Autoimmune Diseases Occur

### 4.1. Periapical Inflammatory Lesions and Root Infections with Systemic Health

The oral cavity and its structures, in particular within the teeth, i.e., the periapical tissues or the alveolar bone, are exposed to constant contact with antigens as a result of their location, and in particular in the pathological state associated with necrotic dental pulp, and they enhance and activate the immune response [86]. The defense reaction includes both specific and non-specific responses leading to the development of inflammation. Too long an inflammatory reaction can damage the periapical bone, which also opens a further path for pathogens, contributing to the even greater development of the inflammatory reaction. Therefore, this type of phenomenon is also reflected not only in the oral cavity but can also affect the systemic state of the body, for example, through the spread of endodontic bacteria or maintaining the local production of soluble forms of regulatory molecules, as a result of recognition by pattern recognition receptors, ingredients produced by pathogens, as well as factors released from damaged cells [86]. An example of such a pathogen is *Candida albicans*, commonly called *C. albicans*, a pathogen capable of colonizing the dentin walls of root canals. It is known to penetrate the dentinal tubules and cause persistent or resistant infections. The concern of *C. albicans* is that, even after intrathecal medications and irrigation solutions have been administered, the fungus can remain in the dental tubules. This can lead to a long-term infection that is challenging to treat [87]. Another bacterial pathogen that causes similar issues is Enterococcus faecalis. This Gram-positive bacterium produces a biofilm in the root canal, which can result in persistent infections within or beyond the roots. What makes this bacterium especially concerning is its ability to adapt to low oxygen levels and nutrient-deficient environments. This allows it to invade dentinal tubules without the support of other bacteria, making it even more challenging to treat [88].

This is because the constant passage of antigens, e.g., bacterial, stimulates the innate immune response by binding to specific receptors, such as Toll-like receptors, which activate intracellular mechanisms to increase cytokine production, such as the NF-κβ pathway [86]. At the same time, cytokines can stimulate the body’s production of acute phase proteins and blood coagulation factors, including CRP and fibrinogen, respectively. Too high a concentration of these compounds may affect their aggregation and accumulation, which may result in thrombosis [86]. Molecular mimicry is another potential mechanism associated with inflammation. It occurs when there is a similarity in the sequence of host proteins and endodontic bacteria, which can activate T or B lymphocytes. This immune response can lead to the development of autoimmunity against these sequences. This process may contribute to the pathogenesis of inflammation and its associated diseases [86].

Epidemiological data indicate that periodontal diseases are one of the most common inflammatory diseases in adults. Studies of root canal pathogens and their role in the development and progression of periodontitis conducted by many specialists have also resulted in a deeper, interdisciplinary look at their involvement in the pathogenesis of systemic diseases such as cardiovascular disease, type 2 diabetes, respiratory infection, neurodegenerative diseases, or cancer [89,90,91].

### 4.2. The Role of Root Canal Infection in the Pathogenesis of Autoimmune Diseases

Taking into account the complexity of interactions between the microbiome of the root canal system, virulent bacterial factors, and the patient’s immune response, it is extremely important to take all measures to limit the development of inflammation, the long-term persistence of which in the human body may lead to systemic defects of the immune system and autoimmune diseases.

#### 4.2.1. Systemic Lupus Erythematosus and Periodontal Disease

Systemic lupus erythematosus (SLE) is one of the systemic connective tissue diseases caused by directing the immune system against one’s own body (this process is called autoimmunity). The disease has many different symptoms and a very diverse course—from quite mild forms, constituting the majority of cases, to severe, even life-threatening forms. The exact cause of systemic lupus erythematosus is not known. It is known that lupus develops in genetically susceptible people when exposed to an additional stimulus, such as exposure to sunlight, bacterial or viral infection, hormonal factors, and even medication [92,93,94,95]. Oral manifestations of SLE are common and typically take the form of painless oral ulcers (oral aphthous ulcers, erosions, hyperkeratosis, and pigmentation) that are often present during exacerbations and are included in the current SLE classification criteria [96,97]. Patients also experience increased dry mouth or reduced salivary flow, affecting oral microbiota [98,99,100]. According to meta-analyses, oral manifestations of SLE are one of the most common and first symptoms of the development of this disease [100]. Although the first research on the link between periodontal disease and SLE was conducted in 1981 [101], the evidence of the link between these diseases still required much intensive and interdisciplinary research [102,103,104,105,106]. These studies showed that patients with SLE had significantly more gingivitis, poor oral hygiene, and a higher rate of periodontal disease than controls in all patient populations tested [102,103,104,105,106]. Moreover, research conducted by Jaworski et al. showed that therapeutic amounts of steroids used to treat SLE may have contributed to the severity of oral disease [104]. Additionally, a comparative study by Novo et al. showed an association between ANCA and periodontitis in patients with SLE [107]. A retrospective case-control study by Wu et al. showed a higher risk of SLE among patients with frequent periodontitis [108]. The presented scientific studies emphasize the need for more frequent dental treatment in patients with SLE due to the high risk of periodontal disease and temporomandibular joint disorders.

#### 4.2.2. Rheumatoid Arthritis and Periodontal Disease

There is evidence that periodontitis, especially oral dysbiosis, is associated with autoimmune inflammatory disease, mainly rheumatoid arthritis (RA). It is a chronic inflammatory disease that affects the joints and various organs. Its most characteristic symptom is pain, stiffness, and swelling of the joints of the hands and feet, but the inflammation can also affect other joints [109,110]. Untreated, the disease most often leads to joint destruction and severe disability, as well as damage to many organs and premature death. The early application of effective treatment inhibits the progression of the disease, prevents its complications, and enables normal functioning. Research indicates that RA is linked to oral disorders, including bacterial and viral infections, gingivitis, periodontitis, or loss of movement in the temporomandibular joint [111,112].

Data in the literature confirm that periodontal disease is even more common in people with rheumatoid arthritis than in healthy people. Patients diagnosed with both diseases are characterized by more severe arthritic symptoms that are more difficult to treat. An unambiguous mechanism underlying the link between these two diseases is not yet fully understood [113]. Researchers suggest the participation of proteins containing citrulline on their surface, which are present not only in the human body (joints) but also among bacteria (especially *Porphyromonas gingivalis*, a common pathogen in periodontal infections). A detailed analysis of this relationship was carried out by Rosenstein et al., which showed that *P. gingivalis* (with peptidyl arginine deaminase activity) is involved in the exposure of people with periodontitis to citrullinated antigens, which, as a result of the development of antibodies against the cyclic citrullinated peptide (CCP), increase the risk of RA [114]. Recent research, conducted by Brewer et al. in 2023, using the latest genetic methods, showed that not only is periodontal disease more common in people diagnosed with RA, but also it is associated with the detection of antibodies against citrullinated proteins (ACPA), which indicates the involvement of oral mucositis in the pathogenesis of RA [115].

Other studies see an important role of the immune system in the connection between RA and periodontitis. This includes the researchers’ demonstrated profiles of host cells and mediators involved in the pathogenesis of chronic periodontitis and RA, which are remarkably similar. In the course of synovitis, leukocyte infiltration, osteoclast activation, and the presence of mast cells as well as T and B lymphocytes, are observed, as well as the involvement of NF-κBligand (RANKL) and TNF-α, whose involvement has also been demonstrated in the course of periodontitis [116,117,118,119].

Despite the differences between RA and periodontal disease emphasized by researchers (RA is an inflammatory autoimmune disease, while periodontal disease is an immuno-inflammatory disease of bacterial origin), there are similarities in inflammatory mediators and other molecular markers in the pathogenesis of both diseases. However, a full understanding of the mechanisms underlying both diseases requires more interdisciplinary research

#### 4.2.3. Sjögren’s Syndrome (SS) and Periodontal Disease

Sjögren’s syndrome is an autoimmune disease in which inflammation and damage to the exocrine glands (mainly lacrimal and salivary glands) develop alongside the simultaneous development of inflammatory changes in many systems and organs [120,121]. The etiopathogenesis of Sjögren’s syndrome is unknown, but genetic factors, possibly also viral infections, play a role in its occurrence [120]. Insufficient salivation, one of the glandular symptoms of Sjogren’s syndrome, causes dry mouth and rapidly progressing tooth decay, which can cause bacteria to penetrate the root canals and develop periodontal diseases [122]. Insufficient saliva, and its incorrect composition, contribute to difficulties in chewing food, its initial digestion and swallowing, and changes in the composition of the microbiome of the entire oral cavity [123,124,125,126]. Due to the characteristic symptoms in the oral cavity, dentists are often the first to detect this disease among their patients. Typically, patients suffer from dry and brittle mucosa, cracked tongue, root, neck, or incisal/tubercular caries, plaque buildup, gingivitis, and/or periodontitis [127,128,129]. Dentists also emphasize that, in the course of Sjögren’s disease, there are also symptoms from the oral cavity, such as recurrent candidiasis, enlargement of the salivary glands, angular cheilitis, changes in the oral cavity resulting from injuries, and difficulties in wearing or maintaining dentures [128,129]. A few scientific studies report that, in patients with Sjogren’s syndrome, despite excellent oral hygiene, there is an increased level of caries accompanied by tooth loss. What is more, there is also a change in the composition of saliva and the protein profile (increase in the level of lactoferrin, beta(2)-microglobulin, sodium, lysozyme C and cystatin C, and a decrease in salivary amylase and carbonic anhydrase), as well as antibodies directed against the Ro 60 and La autoantigens [130,131,132].

#### 4.2.4. Chronic Kidney Disease (CKD) and Periodontal Disease

Research has firmly established that periodontal disease can directly exacerbate kidney dysfunction by triggering inflammation. Studies with animal models have substantiated this by demonstrating significant changes in the renal cortex tissues of those with periodontal inflammation compared to the control group [133]. Furthermore, periodontitis can indirectly impact kidney health by complicating conditions such as obesity and diabetes [133]. Periodontitis has been shown to exacerbate kidney pathology in obese mice on a high-fat diet, with lean mice having increased glucose tolerance compared to mice without periodontitis [133]. Animal model theories seem to apply to patients with chronic kidney disease, as serum urea levels increase with increasing oral pH. In addition, these patients also showed an average lower GFR in patients with oral lesions than in patients without such lesions [133]. Additionally, a correlation has been suggested between periodontitis and increased mortality in patients with chronic kidney disease, with periodontal therapy also suggested to be beneficial for kidney function, possibly by reducing inflammation [133]. Loss of teeth and gums can accelerate in CKD. CKD patients are less likely to receive dental care due to poor oral hygiene. Patients with severe chronic kidney disease often neglect oral hygiene, even when presenting with periodontitis [133].

#### 4.2.5. Potential Pathogenic Link between Periodontal Disease and Inflammatory Bowel Disease

Endodontic bacteria, as well as oral inflammation, are also important for patients with IBD. These types of conclusions were presented by the Imai team [134]. In the study, the researchers assessed the impact of periodontitis on the disease phenotypes of patients with inflammatory bowel disease. The study group included patients with ulcerative colitis [UC] and Crohn’s disease [CD]. In the initial phase of the study, researchers assessed the impact of initial periodontitis on the oral and gut microbiome in the context of inflammatory bowel disease. After 12 months of follow-up, the researchers concluded that, in both UC and CD patients, the gut microbiome was closer to the oral microbiome than the control group. This may suggest that, in patients with IBD, intestinal colonization by oral bacteria is increased. Additionally, these investigators observed that early periodontitis may be associated with worse clinical symptoms in some patients with CD [134].

Another study using an animal model showed a potential relationship between oral cavity inflammation and enteritis exacerbation (Kitamoto’s team [135]). This relationship is related to the Th17 cells that become activated in response to pathogens in the oral cavity. In addition to the fact that these cells have intestinal tropics and migrate to the inflamed intestine, the ingested pathobionts contribute even more to the increase in the inflammatory response, as Th17 cells respond to them again in the intestine. Therefore, this proposed mechanism may contribute to exacerbating intestinal inflammation by increasing the potential inflammatory factors as well as the overactive cells of the immune system [135].

### 4.3. Instrumentation and Irrigation Solutions Manage Endodontic Infections

As previously presented, the bacterial invasion of the dental pulp can lead to inflammation and infection, causing endodontic infections. To manage these infections, root canal treatment is a crucial process that involves instrumentation and irrigation to eradicate the infected tissue and disinfect the root canal system. In this discussion, we will delve into the use of instrumentation and irrigation solutions in the management of endodontic infections [136,137].

During instrumentation, endodontic files are used for mechanical debridement to clean and shape the root canals. The files remove infected pulp tissue and debris, reducing the bacterial load within the canal system. This step allows for better penetration of irrigation solutions. Different techniques, including manual dynamic agitation, ultrasonic activation, or laser activation, can be employed during instrumentation to enhance irrigation effectiveness. These techniques improve the penetration and distribution of irrigation solutions into complex anatomical areas, promoting better disinfection [136,137].

It is important to note that the selection and use of instrumentation and irrigation solutions should be based on the specific clinical scenario, considering the patient’s medical history, the severity of the infection, and the dentist’s professional judgment. The proper disinfection of the root canal system using appropriate instrumentation and irrigation solutions significantly improves the success rate of endodontic treatment by eliminating or minimizing bacterial contamination [136,137].

## 5. Conclusions

The oral cavity is a highly intricate complex environment that handles the intake and processing of food, with teeth playing a crucial role in this process. However, if the protective barriers of the teeth are compromised, opportunistic and harmful microorganisms can infiltrate the root canal system, leading to potential issues such as inflammation, necrosis, and periodontitis. The persistent inflammation at the apical of a tooth or root canal can harm the immediate area and the overall immune system, leading to various health concerns associated with chronic inflammation. People with autoimmune conditions, such as rheumatoid arthritis, systemic lupus erythematosus, or Sjogren’s syndrome, may experience more severe symptoms and have a higher risk of further deterioration of the disease. This is because harmful substances from bacteria and fungi, or local injured tissue, can cause inflammation-causing agents to enter their bloodstream, for example through mouth sores, which could result in increased inflammation and autoimmune reactions.

Therefore, this literature review highlights the critical role of inflammation in the oral cavity, which can significantly impact the entire body in the context of immune system disorders and chronic inflammation. The collected information clearly emphasizes the importance of oral hygiene as an equally important element that can positively affect the patient, especially during systemic treatment.

## Figures and Tables

**Table 1:** Classification of accessory canals by Yoshida et al. [15].

Type	Characteristic
Type 1	Patent accessory canals that communicate with the periodontium and pulpal chamber—‘real canal.’
Type 2	Accessory canals that start in the pulp chamber and end in dentine—‘blind pulp chamber canals.’
Type 3	Accessory canals that start in the periodontium and end in dentine—‘blind periodontal canals.’
Type 4	Accessory canals that start in the pulp chamber, go through dentine, and return to the pulp chamber—‘loop pulp chamber canals.’
Type 5	Accessory canals originating from the periodontium, go through dentine and cementum, and return to the periodontium—‘loop periodontal canals.’
Type 6	Accessory canals found in dentine and cementum, but with no exit—‘sealed dentine canals.’

## Abbreviations

ANCA	Antineutrophil cytoplasmic antibody
CCP	Cyclic citrullinated peptide
CD	Crohn’s disease
CKD	Chronic kidney disease
DAMPs	Damage-associated molecular patterns
GFR	Glomerular filtration rate
LTA	Lipoteichoic acid
MAPK	Mitogen-activated protein kinase
M-CSF	Macrophage colony-stimulating factor
NALP3	NLR family pyrin domain containing 3
NF-κB	Nuclear factor kappa-light-chain-enhancer of activated B cells
NLRs	NOD-like receptors
NOD	Nucleotide-binding oligomerization domain containing
PAMP	Pathogen-associated molecular patterns
PRRs	Pattern recognition receptors
RA	Rheumatoid arthritis
RANKL	Receptor Activator for Nuclear Factor κ B Ligand
SLE	Systemic Lupus Erythematosus
SS	Sjögren’s syndrome
TLR	Toll-like receptor
TLRs	Toll-like receptors
TNF-α	Tumor necrosis factor α
UC	Ulcerative colitis
